# Automatic Inspection of Aeronautical Mechanical Assemblies by Matching the 3D CAD Model and Real 2D Images

**DOI:** 10.3390/jimaging5100081

**Published:** 2019-10-19

**Authors:** Hamdi Ben Abdallah, Igor Jovančević, Jean-José Orteu, Ludovic Brèthes

**Affiliations:** 1 Institut Clément Ader (ICA), Université de Toulouse, CNRS, IMT Mines Albi, INSA, UPS, ISAE, Campus Jarlard, 81013 Albi, France; 2 DIOTASOFT, 201 Rue Pierre et Marie Curie, 31670 Labège, France

**Keywords:** computer vision, manufacturing, maintenance, robotized inspection, handheld inspection, CAD model, viewpoint selection, tracking, matching 3D/2D, augmented reality

## Abstract

In the aviation industry, automated inspection is essential for ensuring quality of production. It allows acceleration of procedures for quality control of parts or mechanical assemblies. As a result, the demand of intelligent visual inspection systems aimed at ensuring high quality in production lines is increasing. In this work, we address a very common problem in quality control. The problem is verification of presence of the correct part and verification of its position. We address the problem in two parts: first, automatic selection of informative viewpoints before the inspection process is started (offline preparation of the inspection) and, second, automatic treatment of the acquired images from said viewpoints by matching them with information in 3D CAD models is launched. We apply this inspection system for detecting defects on aeronautical mechanical assemblies with the aim of checking whether all the subparts are present and correctly mounted. The system can be used during manufacturing or maintenance operations. The accuracy of the system is evaluated on two kinds of platform. One is an autonomous navigation robot, and the other one is a handheld tablet. The experimental results show that our proposed approach is accurate and promising for industrial applications with possibility for real-time inspection.

## 1. Introduction

Industrial quality control is often realized as an automated end-of-line inspection system that checks certain features on the products. In the aeronautical field, many industrial companies use computer vision to automate tiresome operations of quality control in mechanical assembly for several reasons. On one hand, the human operators have implicit limits like overseeing of the missing parts and low repeatability rate [1]. On the other hand, the inspection task must be carried out in a cycle time established for a specific production process which is hard to achieve with manual inspection [2].

In modern manufacturing, industry robots are employed to conduct the inspection tasks with machine vision systems. These robots provide the physical link between intelligence and action. Usually, a robot inspection system has three modules: (1) mechanical: links, joints, end-effector, base, and mobile platform; (2) vision-based sensor system mounted on its end-effector; and (3) the perception, planning, and control unit, which interprets the sensory input and plans and controls the robot’s actions [3].

In this paper, we present a robust approach for detecting a common type of defect on aeronautical mechanical assemblies whose CAD model we dispose and exploit extensively. Our system aims to verify the presence of the correct part within the assembly. It also checks that the part is well mounted. The parts we are checking are metallic supports whose color and texture are often similar to the one of the background, making our task challenging. More details as well as illustrations will be given further in the article.

We evaluated our algorithm using two different systems: robotic and tablet-based handheld interface. Our approach consists of two processes.

First, automatic selection of informative viewpoints is started, given a priori 3D CAD models of the object and of the the whole assembly. The method proposed in this paper aims to find the best viewpoint according to designed scoring function.

Second, after the favorable viewpoints have been selected, our system verifies that the needed elements of the assembly are present and that they have been mounted in the correct position. The CAD model is used as a reference and it describes the assembly as it should be.

The proposed method has the following advantages; it (1) combines the precise information of CAD model with fast and inexpensive 2D sensors, (2) inspects parts with arbitrary geometrical shapes, (3) processes in real-time, and (4) fully automates the inspection process.

This work has been conducted as a successful collaboration between the Institut Clément Ader research laboratory and the Diota${}^{©}$ company (www.diota.com). More precisely, within the Institut Clément Ader research laboratory, the “Metrology, Instrumentation, Control and Monitoring” research group carries out research work on vision-based inspection for manufacturing or maintenance applications. The Diota${}^{©}$ company has been recently focusing on vision-based solutions for industrial control, relying on its core technology in CAD-based tracking and augmented reality.

The remainder of this paper is organized as follows. Section 2 introduces related works. The overview of the proposed inspection system is given in Section 3. The proposed method for the matching between the set of projected edgelets and the set of real edges is explained in Section 4. The overview of viewpoint selection scheme and some considerations about generating a set of candidate local viewpoints and the scoring function are detailed in Section 5. Section 6 shows a few empirical experiments and discusses about experimental results. Finally, in Section 7, some future directions are presented and the paper is concluded.

## 2. Related Work

In the aviation industry, the product inspection requirements have become stricter and more challenging, as products become smaller and more complex and the assembling process becomes faster. Consequently, an automated visual inspection system is one of the most important pieces of equipment for quality assurance and production efficiency with objectivity, endurance, and repeatability equal to and even surpassing human perception.

The systems that we are interested in are based on standard industrial robots that use a vision-based sensor system mounted on their end-effectors, such as those described in [4] for visual weld seam inspection, or the multi-axis Degree of Freedom (DoF) robot arm mounting an optical 3D scanner for inspection of mechanical components. We are also interested in the works aiming to find the optimal viewpoints as the one described in [5]. Further, our focus moves to the developed algorithms for mechanical assembly inspection. We also give some references to the works in the domain of CAD-based tracking and localization, which are not our domains of contribution, but whose results we use as inputs to our inspection method.

In this work, the starting point is a 3D CAD model of an assembly to be inspected. The first step is offline viewpoint selection, which is performed per object to be inspected. In the computer vision community, the viewpoint selection has been investigated. Examples are optimal 3D object recognition [6], grasp detection [7], human actions recognition [8], visual search [9], or global 3D reconstruction [10]. The problem of viewpoint selection is defined as generating candidate viewpoints and constructing scoring function for finding the best viewpoints with the highest score as presented by Liu et al. [11]. For reducing the search space, many authors use an approximate sphere [12] or a cylinder to generate the candidate viewpoints [11]. In our work, the candidate viewpoints are sampled over a sphere, therefore each viewpoint is at a safe distance from the center of mass of the element to be inspected, so as to comply with industrial security standards for ensuring the security of the inspected element as well as of the inspecting robot.

After the offline process of viewpoint selection is completed, the robotic inspection system is ready for operation. Comparison of acquired 2D or 3D data with the CAD model is a fast-growing type of inspection. In fact, it is the only effective and precise method of inspection, because the model contains an exact specification of the part [13] and provides a rich and useful source of information. In early works, Newman et al. [14] presented automated visual inspection (AVI) and techniques that have been reported in the literature from 1988 to 1993. They especially focused on the expanded role of CAD models in many systems of inspection. Jovancevic et al. [15] show that utilizing CAD model whenever possible is beneficial for their results in the task of exterior aircraft inspection. Viana et al. [16] have presented a CAD-based inspection of mechanical parts by means of comparison between a reference and a test image. The CAD-based matching method compares an image generated from a CAD model with an image acquired with the 2D camera, using primitives extracted from the contours. Berndt et al. [17] present a new technique for the detection of assembly errors using virtual images created from the CAD model. Their software compares a virtual image with the image of the real component. VisionLib${}^{©}$, developed by the Fraunhofer IGD, combines the strengths of real-time object localization, live comparison against CAD data, and augmented reality for inspections and virtual measurements. It offers augmented reality-based stationary and mobile inspection systems for quality control. Image processing and a CAD-based approach have been also applied to the shipbuilding inspection task [18].

Recently, systems for aircraft inspection have been developed as well, such as the mobile robot named Air-Cobot [1,15].

Our work strongly relies on a result of CAD-based tracking and localization by combining 3D data with 2D images. Using model-based tracking technologies, CAD data is registered in real-time to objects captured in camera images. De Crescenzio et al. [19] present a prototype system for aircraft maintenance and inspection. The system includes a marker-less camera pose estimation to track mechanical parts by using the SURF features [20] based on an offline and an online process. In the offline process, they first acquire a reference image of the object to be analyzed and they extract local invariant features. These extracted features are stored in the system for later use. In the online process, the system continuously processes the camera’s video stream, processing each incoming frame by extracting the SURF features from the frame and matching with the local features extracted from the reference image.

To the best of our knowledge, the existing methods for assembly/CAD conformity check are based on comparison of rendered synthetic images with real images [16,17]. Those images are further compared in terms of their respective sets of extracted geometrical primitives such as lines and circles/ellipses. Our method proposes three main novelties:

1. Offline phase of 3D edgelets extraction enables the extraction of meaningful 3D edges, which are prone to produce corresponding 2D edges.
2. Offline preparation phase: used for selecting the most favorable viewpoints for our inspection type.
3. Matching edgelets one by one and full control of comparison between expected shape and observed shape by taking into account the environment of the element (context) in both cases, when the element is present and absent.

## 3. The Proposed Inspection Systems

### 3.1. Overview of the Proposed Systems

We are aiming to support two modes of operation: one based on utilizing multiple sensors (two cameras and a 3D scanner) moved by a robot, and another relying on a single camera linked to a handheld tablet.

#### 3.1.1. Robot-Based Inspection

**Figure 1:**
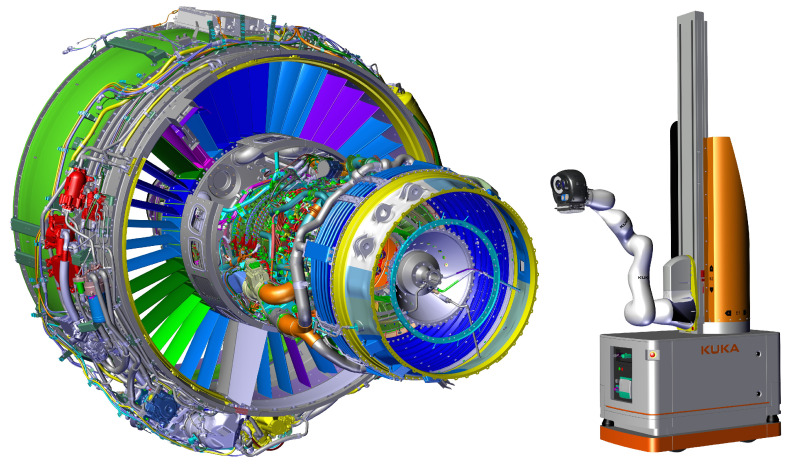
The robot-based inspection platform and an aircraft engine to be inspected.

The robot-based inspection platform is made of a mobile robot (Figure 1) equipped with three sensors (two cameras and a 3D scanner) mounted on the robot arm effector. Note that the sensor DiotaSensor ONE is a product commercialized by Diota${}^{©}$. The same holds for a whole robotic solution we are describing, which is commercialized under the name SIRIS${}^{©}$.

**Figure 2:**
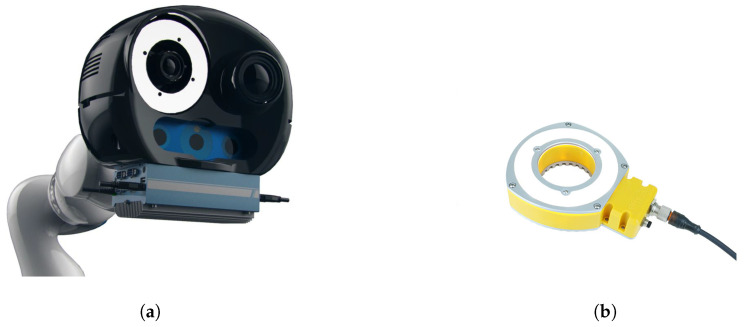
(**a**) Vision-based sensory system mounted on the robot; (**b**) additional lighting around the inspection camera.

The sensors mounted on the robot (see Figure 2) allow several features:

- Inspection: A high-resolution camera with a reduced field of view allows capturing details and observing the elements very finely with 7 DoF. This is a high-resolution camera with large sensor, which, together with 25 mm focal length lens, enables high sensitivity and observability of fine details in the working zone. Our typical working distance is ∼600 mm. At this distance, our image covers a zone 311 mm × 245 mm, and each pixel covers a square of dimensions 0.12 mm × 0.12 mm. Therefore, one-pixel error means an error of ∼1/10 mm. The specifications of the inspection camera are shown in Table 1.
  As shown in Figure 2, our vision-based sensor is equipped with a ring-type Effilux lighting system positioned around the inspection camera. This light source is only activated in the moment of image acquisition, when the robot is not moving.
- Localization: A wide field tracking camera allows precise localization of the effector with respect to the part it controls. The choice of the camera is the result of extensive and permanent evaluation and testing of all significant industrial cameras available on the market (uEye${}^{©}$ (https://en.ids-imaging.com), Basler${}^{©}$ (www.baslerweb.com), etc). Our augmented reality solution (see Section 4.2) is well established and has been accepted by many industrial partners. The specifications of the tracking camera are shown in Table 2.
- 3D scan: A structured light stereo sensor complements the sensor’s capabilities by digitizing the areas observed in 3D.
- Controlling: The whole is controlled by an industrial PC which manages data acquisition and localization of the effector.

In this paper, we focus on the inspection based on 2D image analysis. The preliminary results on the automatic inspection method based on matching the 3D CAD model and real 3D point clouds have been presented in our recent conference paper [21]. These two methods treat two different inspection cases. Although the 2D inspection method presented in this paper puts attention on verifying the presence of certain rigid elements, the 3D inspection method focuses on measuring a 3D distance between nonrigid elements and checking conformity of this distance with predefined security standards.

#### 3.1.2. Tablet-Based Inspection

**Figure 3:**
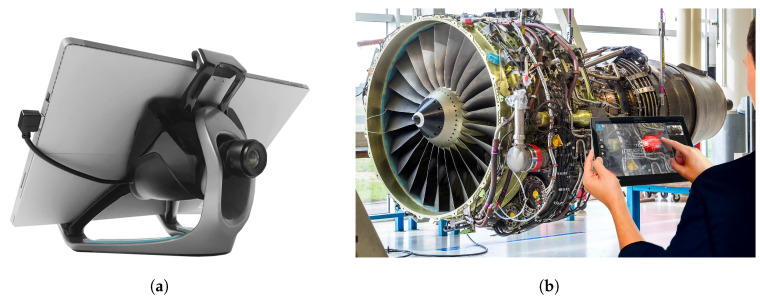
Tablet-based interface equipped with a camera for visual inspection. (**a**) the tablet-based interface equipped with a camera (**b**) the tablet-based handheld interface used by an operator for the inspection of an aircraft engine

The tablet-based inspection platform is made of a tablet with a mounted single camera. This set-up has been commercialized since many years by Diota${}^{©}$ under the name Diota AR Tablet${}^{©}$. The tablet-based interface (Figure 3) is equipped with a HD high-speed industrial camera and ensures a consistent high-quality experience in various work contexts. The device will display 3D model superimposed on a real image of the corresponding item of interest (augmented reality). While the industrial assembly is scanned by an operator, our algorithmic approach automatically checks whether or not all the items have been present and correctly mounted. At the end of the inspection, a report is automatically generated. It includes details of any nonconforming parts that should be replaced or repaired.

### 3.2. Data Acquisition

We use two kinds of platform to collect our data: an autonomous navigation robot (Figure 1) and a handheld tablet (Figure 3). The handheld platform is a tablet computer that utilizes a single camera, whereas the robotic platform utilizes two cameras: one for tracking which has a wide field of view, and one for inspection which has a smaller field of view. By independently and separately using both platforms, to test the robustness of our approach, we collected data indoors with different points of view (angle, scale) and with different lighting conditions.

**Figure 4:**
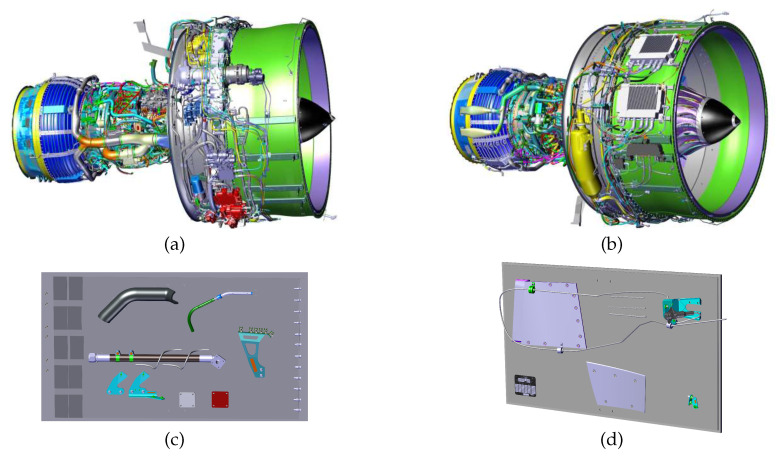
Some of our CAD models: (**a**,**b**) aircraft engine, (**c**,**d**) two testing plates with several elements.

**Figure 5:**
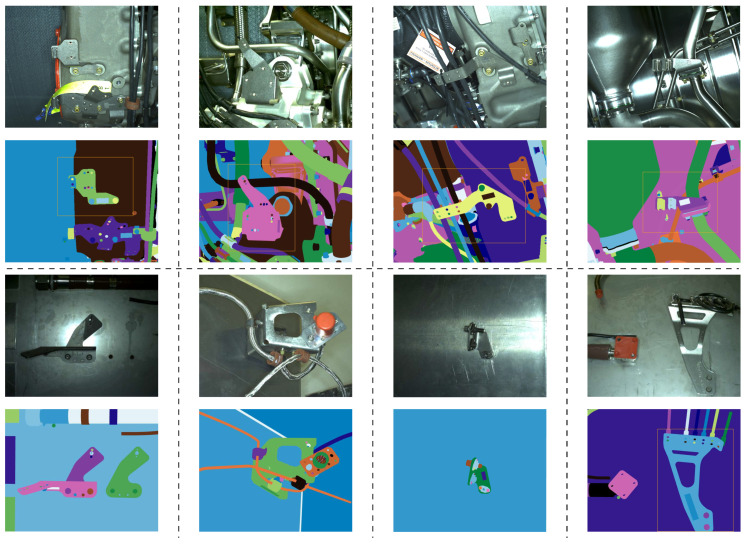
Examples of our dataset: (1st and 3rd rows) real images and (2nd and 4th rows) corresponding renders of CAD models.

The data collected from the tablet is meant for real-time use, therefore images as well as videos are collected. The data from the robotic platform is meant for the analysis with no strong time constraints, as it can be done during and after the data collection routine is completed by the robot. A few examples of our CAD models and data samples are shown in Figure 4 and Figure 5, respectively.

## 4. Inspection Process for the Defect Detection

**Figure 6:**
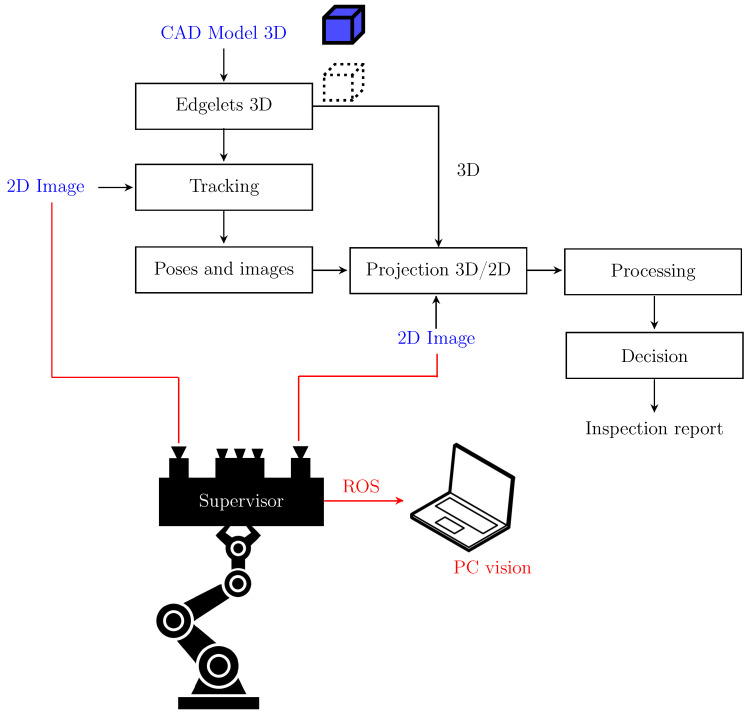
Overview of the detection phase during online processing using robot-based inspection.

**Figure 7:**
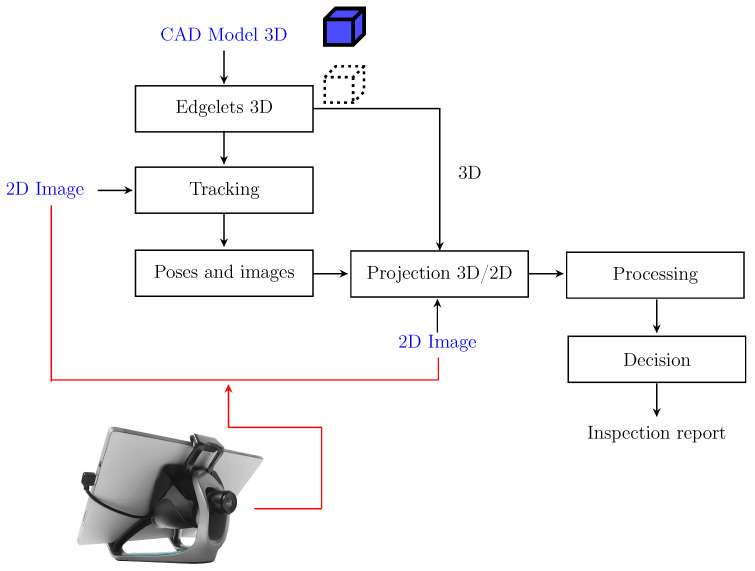
Overview of the detection phase during online processing using tablet-based inspection.

Our inspection process for the defect detection, illustrated in Figure 6 and Figure 7, consists of four main steps. First, from the CAD model of the observed object, 3D contour points (edgelets) are extracted. Further, the edgelets with their direction vectors are projected onto the image using a pinhole camera model (extrinsic and intrinsic parameters). Note that the pose of the effector is due to the localization module. Finally, we perform the matching between the set of projected edgelets and the set of real edges. We explain each building block of our strategy in the following sections.

### 4.1. Step 1 (Edgelet Generation)

**Figure 8:**
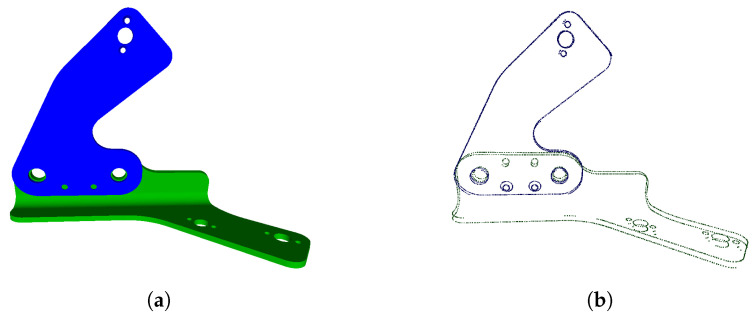
Edgelets extraction: (**a**) example of CAD part, and (**b**) edgelets extracted from this CAD part.

An edgelet, $E_{i}$, is defined as a 3D point, $p_{i}=\left(x_{i},y_{i},z_{i}\right)$, and a normalized direction vector, $\vec{d_{i}}$, of the edgelet. These points, $p_{i}$, represent the 3D contour points of an object, see Figure 8. The edgelets are extracted offline by rendering a CAD model, as explained in [22]. The edgelets has been used in other works, see, e.g., in [23,24,25].

The edgelet generation process is well explained in [22]. Loesch et al. [22] introduce an improvement of this process through “dynamic edgelets” extracted online for each point of view. It should be noted that we have opted for using “static edgelets” defined in [22], as edgelets extracted offline using the dihedral angle criteria. This is because, as mentioned by [22], the generation of dynamic online edgelets induces an additional computing cost. Moreover, we use the extracted edgelets for generating optimal views in an offline process.

- In the case of robot, there is no interest for dynamic edgelets, as the point of view is known in advance and there is neither video stream nor tracking as in the case of tablet.
- In the case of tablet, we have more severe limitations in computing power so the static edgelets are our first choice for the moment.

The output of this step is a set of edgelets that are evenly distributed.

### 4.2. Step 2 (Camera Pose Estimation)

Advantage of our use case is that we dispose of the CAD model of the assembly. Further, we rely on an in-house developed system for initial pose estimation based on 2D-3D alignment, our inspection camera being then considered as moving in a partially known environment. The mentioned algorithm accurately estimates the camera’s pose with respect to the object by including the geometric constraints of its CAD model in an optimization process. In other words, the object is tracked in real time.

### 4.3. Step 3 (3D/2D Projection)

**Figure 9:**
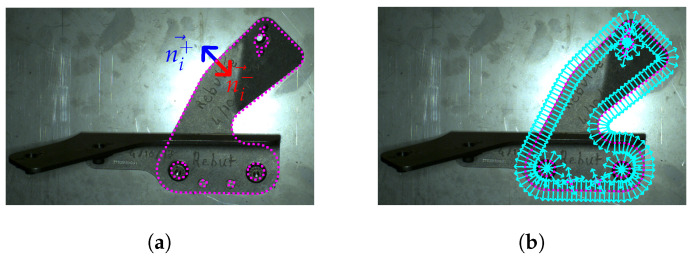
(**a**) Projection of edgelets (3D points $p_{i}$), and (**b**) inward-pointing normals, $n_{i}^{+}$, outward-pointing normals, $n_{i}^{-}$, and generation of search line $l_{i}$.

In this phase, we project 3D points of edgelets onto the image using known extrinsic and intrinsic camera parameters (see Figure 9). The input of this step are an image, the camera pose with respect to the assembly (CAD), and a set of evenly distributed edgelets of the element to be inspected.

To acquire the camera intrinsic parameters, we used an offline calibration method based on the observation of a classical pattern. Based on various observations of the pattern under different arm configurations, we obtained the intrinsic parameters of all the cameras used on the system along with the extrinsic parameters (relative localization) and their absolute localization relatively to the arm effector reference frame. A detailed description of the calibration procedure is beyond the scope of this paper.

Having the pose and camera intrinsic parameters, we can project the 3D point $p_{i}$ (see Figure 9) and the direction vector $\vec{d_{i}}$. By rotating the projected direction vector $\vec{d_{i}^{p}}$ by $+90^{\circ }$ and $-90^{\circ }$, we obtain the inward-pointing normal, $n_{i}^{+}$, (pointing towards the interior of the surface) and outward-pointing normal, $n_{i}^{-}$, (pointing towards the exterior of the surface). Therefore, following is true; $∡\left(\vec{d_{i}^{p}},n_{i}^{+}\right)=+90^{\circ }$ and $∡\left(\vec{d_{i}^{p}},n_{i}^{-}\right)=-90^{\circ }$. By concatenating $n_{i}^{+}$ and $n_{i}^{-}$, we obtain search line $l_{i}$ (see Figure 9b).

### 4.4. Step 4 (Matching Projected Edgelets with Real Edges)

The goal of this step is the matching between the set of projected edgelets and the set of real edges according to the direction of the normal vectors calculated in step 3. We calculate real edges by employing known Canny edge detector [26]. If at least one real edge is found on the search line, the edgelet is considered matched; otherwise, it is considered not matched.

#### 4.4.1. Parasitic Edges Handling

**Figure 10:**
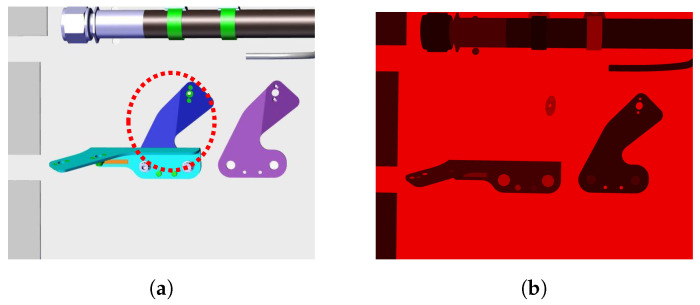
(**a**) Example of CAD model; (**b**) context image of the blue element.

**Figure 11:**
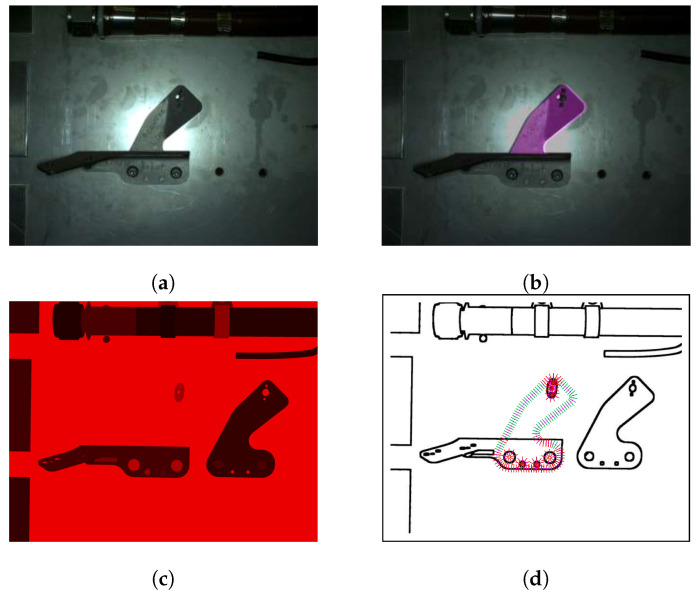
Illustration of the parasitic edges handling: (**a**) input image, (**b**) projection of the element to be inspected, (**c**) context image of the element to be inspected and its projected edgelets, and (**d**) gradient changes in the context image and considered edgelets (green) and rejected edgelets (red).

In this section, we describe our principal contribution, which was introduced to remove as much as possible irrelevant edges. Namely, we anticipate the existence of some portion of parasitic edges coming from other elements mounted in the vicinity of the inspected element. Indeed, often there are edges very close to the external boundary of the element to be inspected. We call them *parasitic edges* or unwanted (irrelevant) edges. To solve this kind of problem, we introduce a new notion called context image of an element to be inspected (see Figure 10). From the CAD data and the particular camera position, we form a synthetic view (context image), which has exactly the same perspective as the real image, only that the inspected element is excluded from the assembly (see Figure 11).

The process of decreasing number of parasitic edges is illustrated in Figure 11. First, we project the edgelets of the element of interest onto the context image, as explained in the step 3.

Further, we form search lines (see Figure 11d) and look for the intensity change in this virtual context image. Intensity changes are shown in Figure 11d. If such a change in context image is found, it is very probable that this edge will be present in the real image as well. Therefore, we do not consider this edgelet for matching. These edgelets are shown in red in Figure 11d. Other edgelets, shown in green in Figure 11d, are considered in matching phase.

#### 4.4.2. Edges Weighting

**Figure 12:**
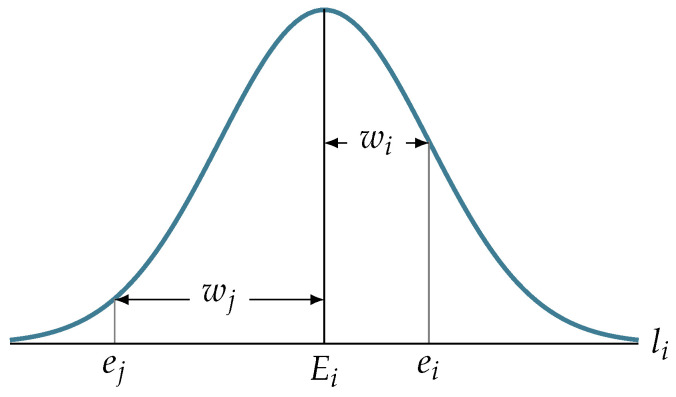
Illustration of edges weighting.

For each edgelet in the 3D CAD projected onto the image plane, we are interested in finding an image location that contains a real change in image intensity (a real edge). For this, we first produce an edge image and search for candidate locations along a search line, $l_{i}$, that is obtained as described in step 3. Each edge location along the search line that is within a prespecified distance from the location of the projected edgelet is weighted according to a Gaussian function of the distance of the edge point from the location of the projected edgelet. $W_{i}$ is a weight of the edge $e_{i}$, and $W_{j}$ is a weight of the edge $e_{j}$ (see Figure 12). Instead of simply counting matched edgelets, these weights will be added up in the final score. We do this to favorize the edgelets matched with very close real edges and penalize those matched with real edges that are far away.

#### 4.4.3. Gradient Orientations

**Figure 13:**
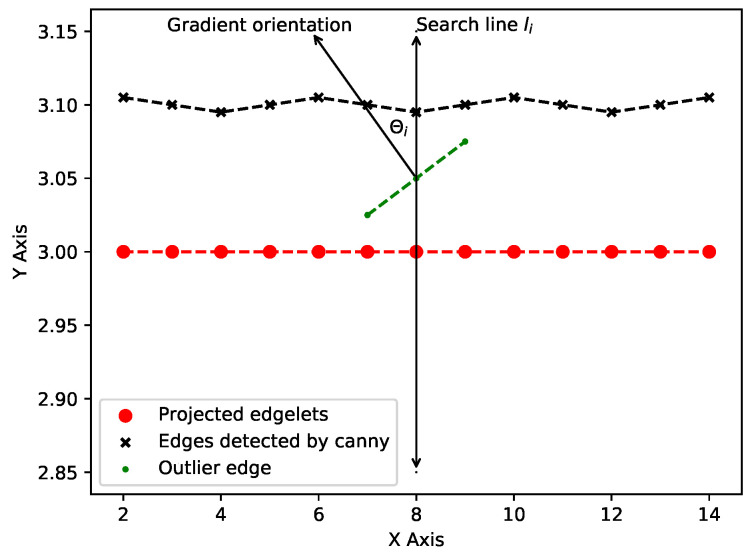
Challenges due to outlier edges, such as those due to image noise and edges arising from features that do not belong to the inspection element, when searching for an image edge corresponding to the projected edgelet.

When searching for an edge in the image, we may encounter edges because of image noise or geometric features that arise from the parts different from the inspection element. To reject such candidates, at each encountered edge location, we compute gradient of the edge and find the angle it makes with the search line $\theta_{i}$ (see Figure 13). If this angle is greater than a threshold ($\theta_{i}$>$\theta_{th}$), we reject the candidate and continue our search. If no edges are found within the search limits, the orientation criteria are satisfied, and we decide that no edge corresponding to the inspection element of interest is present. In our experiments, we have set $\theta_{th}=20^{\circ }$.

#### 4.4.4. Occlusion Handling Using CRI (Color Rendering Index)

**Figure 14:**
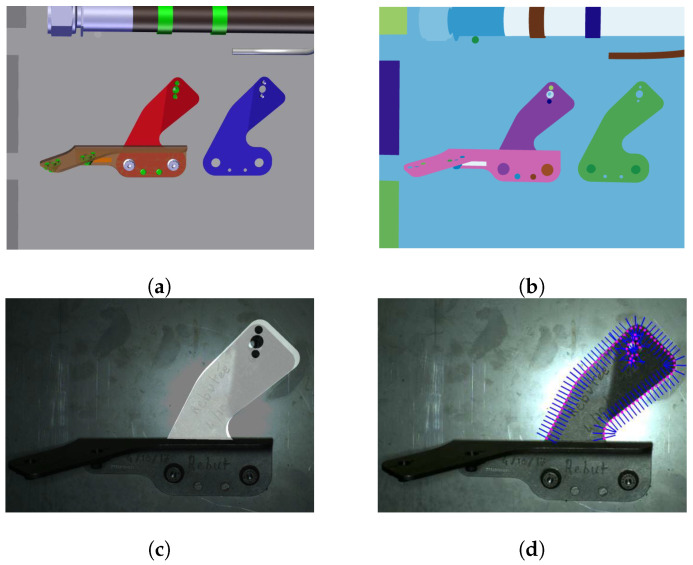
Occlusion handling by rendering using Color Rendering Index (CRI): (**a**) CAD model rendered with CATIA composer, (**b**) CAD model rendered using CRI, (**c**) mask generated using CRI render, white areas represent region to be inspected, and (**d**) filtered edgelets (Figure 11d) projected inside the region of interest specified by the generated mask.

Image locations where edgelets of an inspection element are projected can belong to a different element, due to the inspection element being occluded by it in the real assembly. Searching for real image edges corresponding to the projected edgelets at such image locations is thus incorrect. An example of an occlusion problem is shown in Figure 14. In Figure 14, we find that the red element is behind the transparent green element. This means that part of the red element should be occluded by the transparent green element.

Occlusions are managed by determining what objects are in front of others in a scene, which allows determining whether each rendered object appears in front of any other object in the scene, on a per-pixel basis.

To ignore such locations, we first render the inspection element along with the surrounding context by assigning to each element a different random color using the Color Rendering Index (CRI). To filter projected edgelets occluded by another elements, we generate a mask using the colored render, so that all areas in the render that have a color different from that of the inspection element are ignored. This way, only non-occluded edgelets are considered for matching.

In computer graphics, there are two popular rendering techniques for handling occlusions: Z-buffer technique and classic ray tracing.

The Z-buffer (or depth Buffer) is intrinsically used by the classical graphic pipeline, moreover the Z-Buffer only offers a finite amount of precision to store the depth value for each pixel, problems can occur when multiple objects resolve to the same depth value. In addition, the available precision is affected by the choice of the near and far clipping planes for the 3D scene [27].

Ray tracing is also an effective rendering technique, but it is very slow and is massively used for collision checking on some sparse pixels and knowing which the object are collided (we need all the image here and do not need to know which object is hided by the one in front). Moreover, none of the two mentioned technique will add quality to the mask rendering.

The proposed CRI technique is perfectly adapted to the objectives: knowing which object can be seen at which pixel of the image. In addition, classical graphic rendering with using flat color as index allows to do it very quickly.

The operations described from Section 4.4.1, Section 4.4.2, Section 4.4.3 and Section 4.4.4 provide the so-called filtering of projected edgelets.

### 4.5. Step 5 (Making Decision on State of the Element)

Producing an inspection decision based on matching filtered edgelets independently, with edges in the real image, can lead to false positives, as the matched edges in the real image may also arise from an element with a different shape than the inspected one. To take the global shape of the element into account, we propose to characterize the filtered edgelets and the edges detected in the real image using the Shape Context framework [28]. The Shape Context (represented by a set of points sampled from the edges of the object) is a shape descriptor that captures the relative positions of points on the shape contours. This gives a globally discriminative characterization of the shape, and it is not just a localized descriptor. These are then used to measure similarities between shape context representations of the two sets of edge locations.

**Figure 15:**
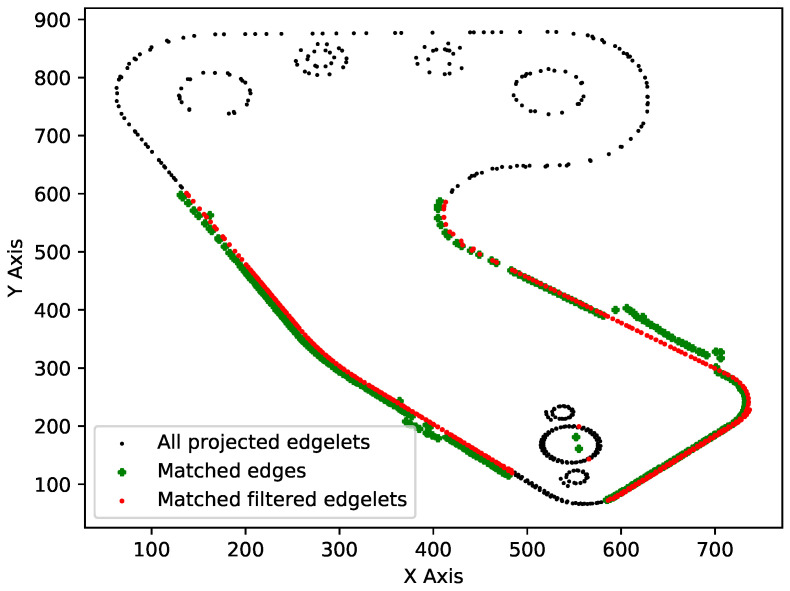
The shapes used in the shape context approach.

For example, Figure 15 shows all projected edgelets (black), matched edges (green), and filtered projected edgelets (red). In this example, the computation of the distance between the green shape and the red shape provides a ${\mathrm{similarity}}_{\mathrm{score}}$ = 0.01.

**Figure 16:**
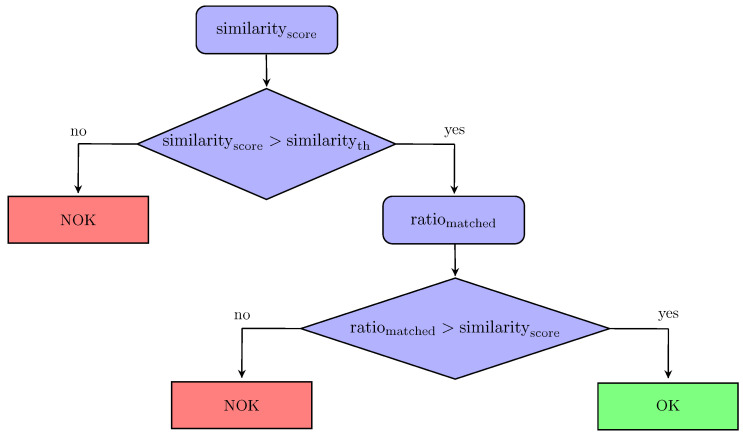
Decision-making.

The workflow of the making decision step is shown in Figure 16. The decision is taken using two indicators: the similarity score (${\mathrm{similarity}}_{\mathrm{score}}$) and the matched ratio (${\mathrm{matched}}_{\mathrm{ratio}}$), represented by the relationship between matched and not matched edgelets.

**Figure 17:**
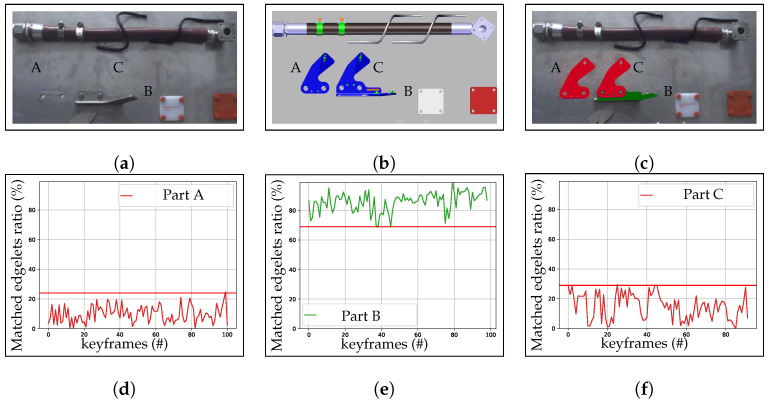
(**a**) CAD model; (**b**) input image; (**c**) result of inspection with green OK (element present) and red NOK (element absent or incorrectly mounted); (**d**–**f**) the ${\mathrm{matched}}_{\mathrm{ratio}}$ (curve) and threshold ${\mathrm{matched}}_{\mathrm{th}}$ (red horizontal line), respectively, computed on the three different cases: element A (absent) in red, element B (present) in green, and element C (incorrectly mounted) in red.

In Figure 17, we show the ${\mathrm{matched}}_{\mathrm{ratio}}$ and ${\mathrm{matched}}_{\mathrm{th}}$ computed on two different cases (element OK and element NOK).

- If ${\mathrm{similarity}}_{\mathrm{score}}<{\mathrm{similarity}}_{\mathrm{th}}$, the element is considered NOK (absent or incorrectly mounted). In our experiments, we set ${\mathrm{similarity}}_{\mathrm{th}}=0.7$, based on an extensive comparison between different shapes.
- If ${\mathrm{similarity}}_{\mathrm{score}}>{\mathrm{similarity}}_{\mathrm{th}}$, we compute the ratio between the number of matched edgelets and the number of not matched edgelets (${\mathrm{matched}}_{\mathrm{ratio}}$).
- If ${\mathrm{matched}}_{\mathrm{ratio}}>{\mathrm{matched}}_{\mathrm{th}}$, the element is considered conform; otherwise, the element is considered as defective (missing or badly mounted). The value of ${\mathrm{matched}}_{\mathrm{th}}$ has been determined experimentally (see Section 6).
- In conclusion, the decision is taken using two indicators: the similarity score (${\mathrm{similarity}}_{\mathrm{score}}$) and the matched ratio (${\mathrm{matched}}_{\mathrm{ratio}}$).

## 5. Viewpoint Selection

The initial set-up of camera viewpoints cannot be done manually, because a human operator cannot define with sufficient accuracy the camera position that will allow acquiring the best viewpoint of the element to be inspected. Therefore, we need a a (semi-)automatic offline configuration process that is used to compute the best viewpoints, which can help to improve the quality and the efficiency of inspection [29].

**Figure 18:**
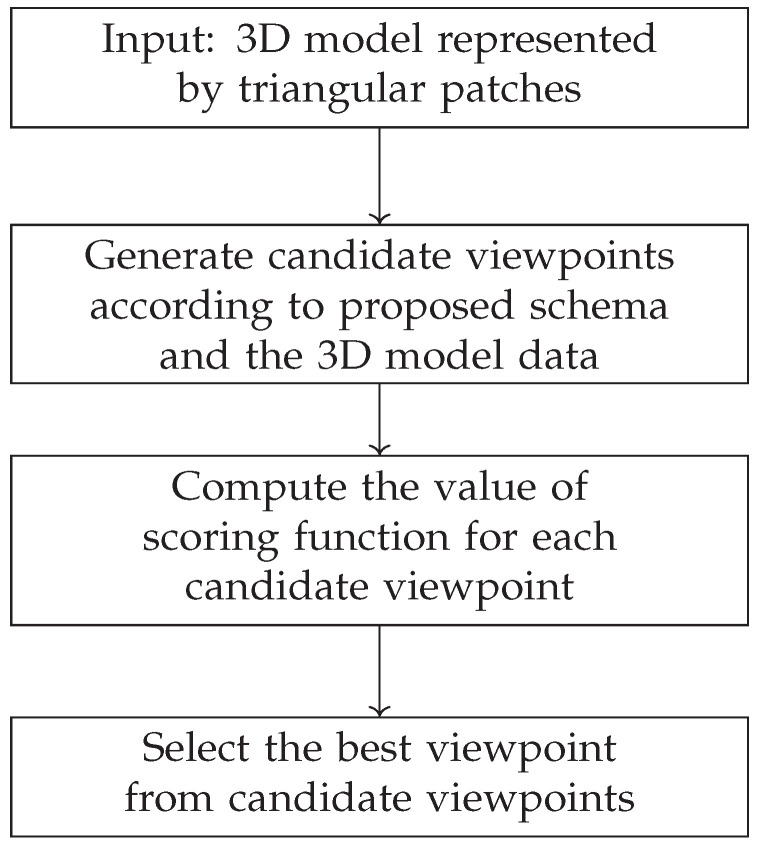
The overview of viewpoint selection scheme.

The strategy proposed to find the best viewpoint (computing the 6D location (position and orientation) of the camera with respect to the scene to be observed) for the aeronautical mechanical assembly inspection, based on the 3D model of the assembly, is illustrated in Figure 18. A scoring function is constructed which combines three factors including the amount of the information, the viewing quality, and the visibility of the information to evaluate the quality of the candidate viewpoint [11]. Finally, the best viewpoint can be selected from all of candidate viewpoints according to the scoring function.

### 5.1. Generate a Set of Candidate Viewpoints

A viewpoint, $V_{point}$, is defined by a point, V(x,y,z), around an approximate sphere and the sensor positioning (distance and orientation) relatively to the part surface. This sphere is defined as a set of views.

**Figure 19:**
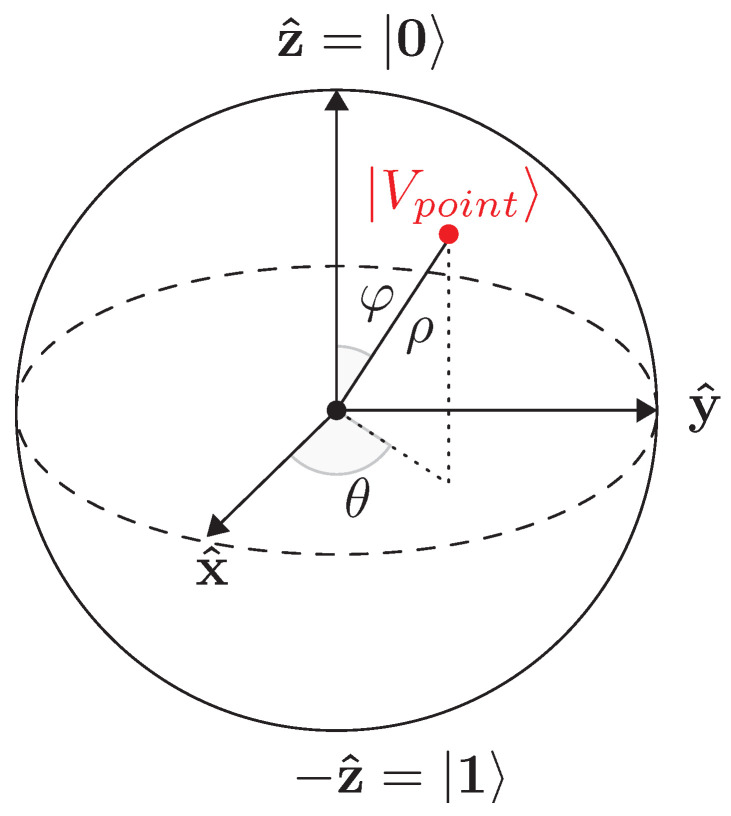
A viewpoint on the visibility sphere.

The radius, ρ, of the visibility sphere (Figure 19) is chosen such that each viewpoint is at a safe distance from the center of mass of the element to be inspected $\left(c_{x},c_{y},c_{z}\right)$. This is to comply with industrial security standards for ensuring security of the inspected element as well as of the inspecting robot.

**Figure 20:**
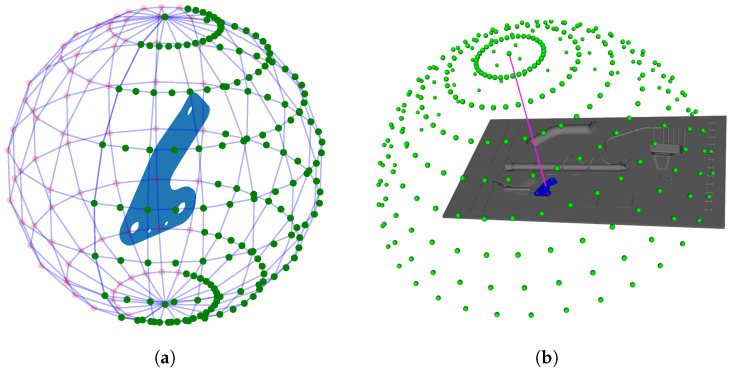
The candidate viewpoint region is defined as a part of sphere surface. The accessible viewpoints in green and the inaccessible viewpoints, due to occlusions related to the global CAD, in red (**a**) for a given element to be inspected and (**b**) for the whole assembly.

The sphere (Figure 20) is defined as a set of viewpoints, and the candidate viewpoints are evenly distributed on the sphere surface according the range of longitude, θ, and colatitude, φ.

### 5.2. Selection of the Best Viewpoint from Candidate Viewpoints

The best viewpoint is selected according to the value of the scoring function. The scoring function $f_{score}$ quantifies the quality of the candidate viewpoint. The best viewpoint with highest score is selected. Our scoring function combines three criteria:

– Visibility of the information, $f_{visibility}$.
– Ambiguity of observed shape due to parasite edges, $f_{parasite}$.
– Similarity of observed shape to expected shape, $f_{shape}$.

#### 5.2.1. Visibility of the Information $f_{visibility}$

We evaluate each generated viewpoint for visibility of the inspection element by taking into account the occlusion using CRI as explained in Section 4.4.4.

**Figure 21:**
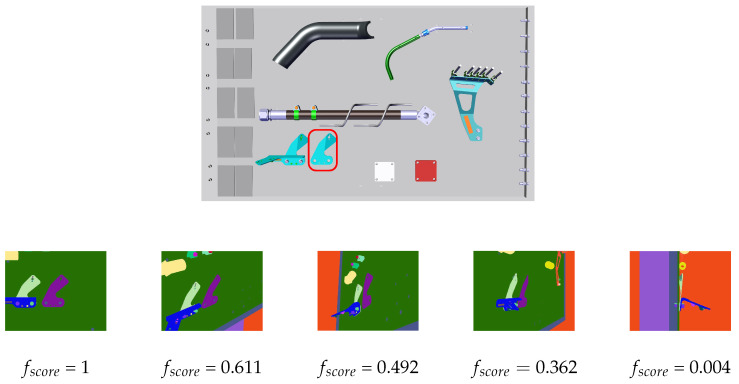
(1st row) CAD model and (2nd row) different candidate viewpoints for inspecting the element in the red rectangle, with the corresponding $f_{score}$.

The viewpoint that yields a render with the greatest number of pixels belonging to the inspection element is chosen to be the viewpoint with best visibility, and therefore the best point of view for performing the inspection task according to this “visibility” criteria (see Figure 21).

#### 5.2.2. Ambiguity of Observed Shape Due to Parasite Edges $f_{parasite}$

Each candidate viewpoint is also evaluated by computing the number of parasite edges and edgelets of the inspection element in a render from the viewpoint as described in Section 4.4.1.

**Figure 22:**
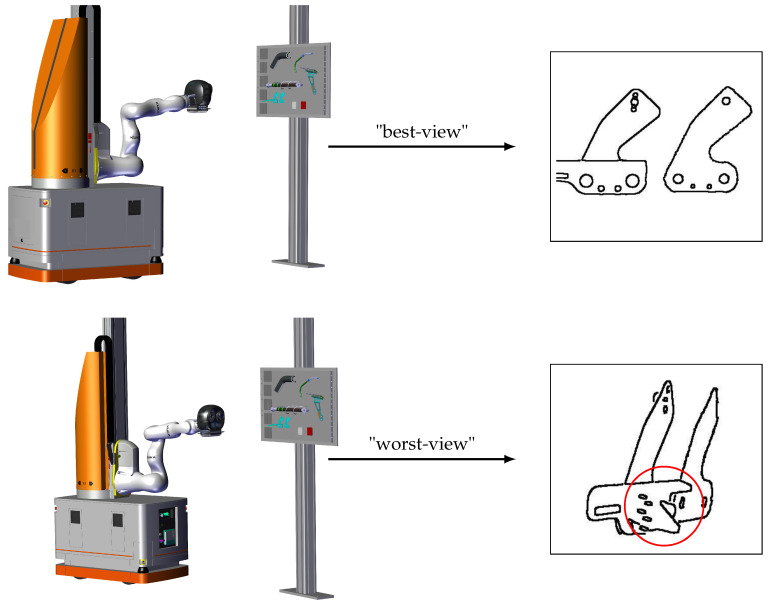
Two viewpoints with best-view and worst-view according to the number of parasite edges.

The viewpoint that maximizes the ratio of the number of edgelets after filtering to the number of parasite edges is chosen as the best inspection viewpoint according to this criteria. In Figure 22, the difference between a viewpoint that yields many parasite edges and a viewpoint that yields few is presented.

Note that there is no guarantee that any viewpoint will be chosen for a given element, namely, if too few edges are left, we announce not being able to perform inspection.

#### 5.2.3. Similarity of Observed Shape to Expected Shape $f_{shape}$

**Figure 23:**
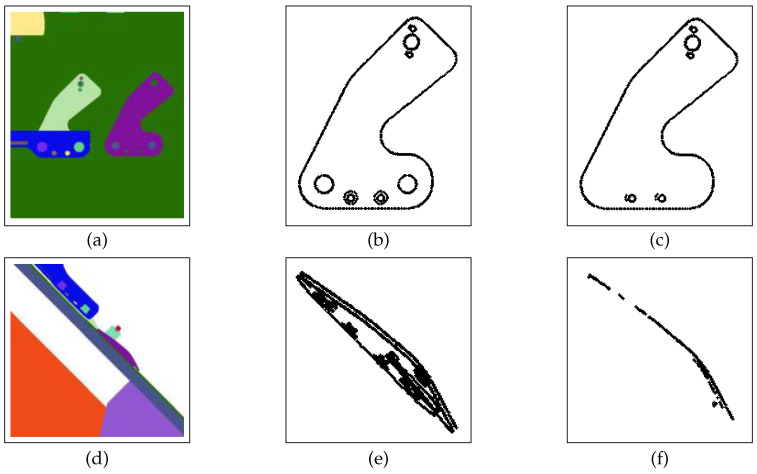
(**a**) “Best-view”, (**b**) all edgelets of best-view, (**c**) filtred edgelets of best-view, (**d**) “worst-view”, (**e**) all edgelets of worst-view, and (**f**) filtered edgelets of worst-view.

Finally, each candidate viewpoint is alsot evaluated by computing the similarity between the shape of filtered projected edgelets of the inspection element (obtained by rejecting those prone to parasitic edges and occlusion) and the unfiltered projected edgelets of the same, i.e., the expected shape (see Figure 23).

#### 5.2.4. The Scoring Function $f_{score}$

The three criteria presented before for evaluating each viewpoint are combined using a scoring function $f_{score}$ detailed below,

$$f_{score}=w_{v}\times f_{visibility}+w_{p}\times f_{parasite}+w_{s}\times f_{shape}$$

$$w_{v}+w_{p}+w_{s}=1$$

$w_{i}$’s are weights that assign importances to the different viewpoint evaluation criteria, and they can be adjusted by the user depending on the importance of the criteria for a specific inspection application. $f_{visibility}$, $f_{parasite}$, and $f_{shape}$ are normalized.

#### 5.2.5. Viewpoint Selection in the **Handheld** Tablet Mode Based on Augmented Reality

**Figure 24:**
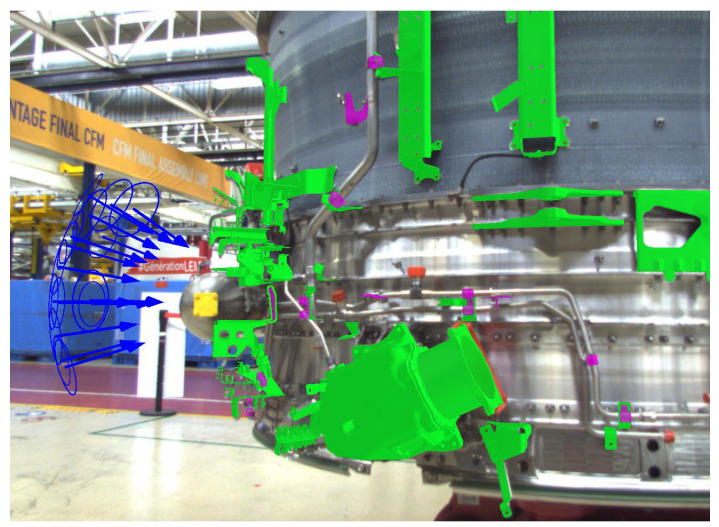
User guidance in the handheld tablet mode (see the blue arrows).

Viewpoints are generated in an offline process by using the CAD model of the assembly. While in the robot mode, the robot is programmed to reach as close to those points as possible (path planning); in the tablet mode, there is an augmented reality-based graphical interface which guides the operator to reach the desired viewpoints or at least their neighborhoods (see Figure 24). Note that our CAD-based tracking module enables the augmented reality experience and constant real-time pose estimation of the camera with respect to the inspected assembly.

## 6. Experiments and Discussion

### 6.1. Illumination Description

Our work took inside a building/factory, which allows a quite stable environment/stable light conditions. Moreover, for the inspection camera, we can adjust the exposure depending on the object to inspect. Either we give a constant exposure time or choose a level of exposure, which will be set by an automatic exposure algorithm. Our robot is not moving when acquiring images. Therefore, we are taking more than one acquisition for each element. We change the exposure time between those acquisitions. The goal is to improve as much as we can the contrast of the image by choosing the one which has the most favorable histogram within region of interest. Depending on the element and the inspection task, this region of interest can be either the whole image either the part of it. Additionally, to the exposure time of the camera, we are also controlling the additional light source that we added around our inspection camera. We are activating this light source ONLY in the moment of image acquisition, when the robot is not moving.

Our CAD-based tracking module, which exploits the tracking camera images, is a robust stable solution already industrialized for years. We do not use any additional light source for this task, as artificial light can cause problems in performing tracking. We are relying on the ambient light coming from the context of the factory.

### 6.2. Performance of Tracking

In terms of performance, the reprojection error of the assembly edges is 1.5 pixels. Our use case is conformity of the assembly with CAD (missing elements), and not precise measures of the surface defects, etc. Therefore, 1.5 pixels is sufficient for our task.

Usually the elements which are inspected make a minority of the elements observed by the tracking camera. The tracking module works with at least 40% of the CAD present in reality. In our use case (typically assemblies close to the end of the production line), this is always satisfied.

### 6.3. Study of Some Parameters

First, we conducted experiments to determine the effect of parameters on the inspection accuracy and also to obtain the best parameters for each type of data. Parameters used in this exploration are (a) threshold (${\mathrm{matched}}_{\mathrm{th}}$) for making decision on state of element, and (b) the high $t_{h}$ and low $t_{l}$ threshold used by the hysteresis procedure of Canny edge detection algorithm.

#### 6.3.1. Determination of Optimal ${\mathrm{matched}}_{\mathrm{th}}$

To find the optimal decision threshold, we conducted some experiments to compute the optimal parameter of our algorithm and evaluate its effectiveness on six inspected elements with a total number of 541 images acquired indoors at different scale, angle, and lighting conditions.

**Figure 25:**
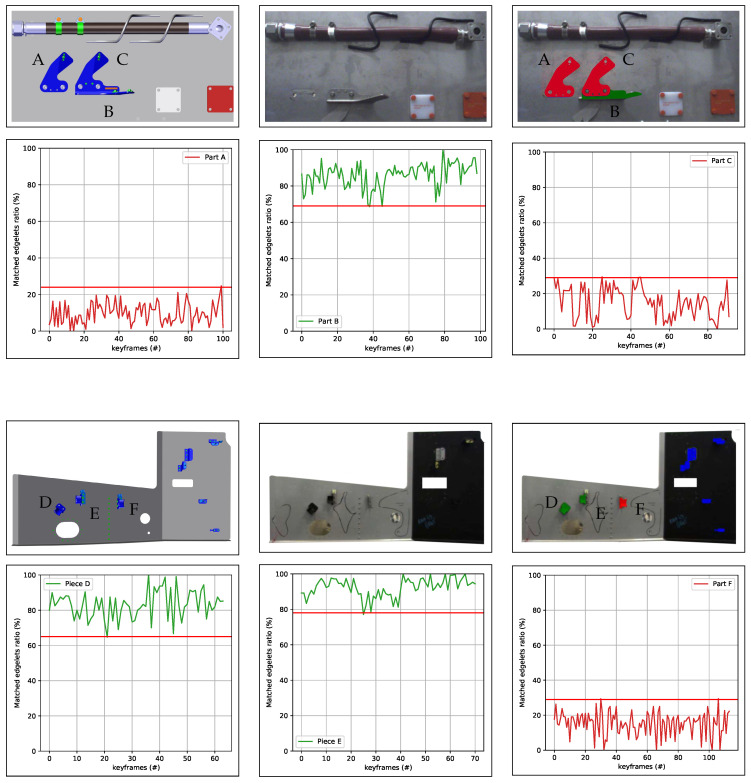
Experimental results for determining the optimal $ratio_{th}$. The red horizontal line represents the min matched ratio in the case OK (element in green) and the max matched ratio in the case NOK (element in red).

According to the results obtained in Figure 25 and Table 3, we can use ${\mathrm{matched}}_{\mathrm{th}}$ in the range of {30%, 60%}. In most cases, we set ${\mathrm{matched}}_{\mathrm{th}}=40\%$.

#### 6.3.2. Determination of Optimal Canny Edge Detection Parameters

**Figure 26:**
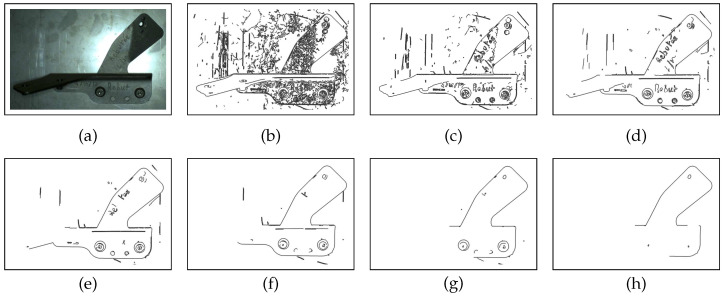
Images of Canny algorithm results (**a**). Images (**b**–**h**) have been obtained using the high and low threshold values specified in Table 4.

Canny edge detection contains two adjustable parameters—high, $t_{h}$, and low, $t_{l}$, thresholds—for the gradient magnitude image, which can affect the effectiveness of algorithm. The number of edge points is dependent on the choice of these thresholds. Figure 26 shows the influence of these values on edge detection. In our experiments, we used $t_{l}$ in the range {20, 40}. Following Canny’s recommendation, the high threshold should be three times the lower threshold ($t_{h}=3\times t_{l}$). In most cases, we set $\left(t_{l},t_{h}\right)=\left(30,90\right)$. When we reduce the lower threshold, the algorithm gives more edge points, which produces more noise (see Figure 23b). We reject noise edges by employing our gradient orientation condition described in Section 4.4.3.

After the optimal parameters of the algorithm have been determined, we can evaluate our algorithm. The proposed inspection method has been tested on two kinds of platforms: a handheld platform and a robotized platform.

The framework used for the evaluations is first provided in Section 6.4.

### 6.4. Accuracy, Sensitivity, Specificity

Table 5 outlines definitions of the true positive (TP), the false positive (FP), the true negative (TN), and the false negative (FN) in defect detection [30].

Based on these notations, the detection success rate (known also as detection accuracy) is defined as the ratio of correct assessments and number of all assessments. Sensitivity is defined as the correct detection of defective samples. Similarly, the specificity can be defined as the correct detection of defect-free samples. Finally, the overall accuracy could be calculated based on the metrics of sensitivity and specificity. The accuracy, sensitivity, and specificity can be defined as

$$\mathrm{accuracy}=\frac{\mathrm{TP}+\mathrm{TN}}{\mathrm{TP}+\mathrm{FN}+\mathrm{TN}+\mathrm{FP}}$$

$$\mathrm{sensitivity}=\frac{\mathrm{TP}}{\mathrm{TP}+\mathrm{FN}}$$

$$\mathrm{specificity}=\frac{\mathrm{TN}}{\mathrm{TN}+\mathrm{FP}}$$

#### Precision and Recall

Precision and recall metrics are used to validate the performance of defect detection. Precision and recall can be defined as

$$\mathrm{precision}=\frac{\mathrm{TP}}{\mathrm{TP}+\mathrm{FP}}$$

$$\mathrm{recall}=\frac{\mathrm{TP}}{\mathrm{TP}+\mathrm{FN}}$$

$$F_{score}=2\times \frac{\mathrm{precision}\times \mathrm{recall}}{\mathrm{precision}+\mathrm{recall}}$$

The $F_{score}$ is calculated to measure the overall performance of a defect detection method. The highest value of the $F_{score}$ is 100%, whereas its lowest value is 0%.

### 6.5. Experiments Using the Handheld Inspection Platform

The performance of the proposed method was evaluated by considering the performance metrics such as accuracy, sensitivity, specificity, precision and recall defined in Section 6.4.

**Figure 27:**
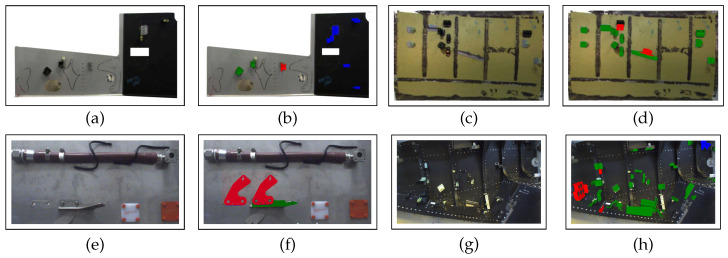
Dataset used to evaluate our algorithms: (**a**,**c**,**e**,**g**) real images and (**b**,**d**,**f**,**h**) corresponding inspection results with OK elements in green and NOK elements in red.

We performed 4 experiments with a total number of 74 inspected elements in 7256 images. A few examples of our dataset are shown in Figure 27. The experiments show that our method can run in real-time on a video-stream with HD resolution (1920×1080). The values of specificity, accuracy, sensitivity, precision and recall of our method are shown in Table 6 and Table 7.

### 6.6. Experiments Using the Robotized Inspection Platform

The robotic platform utilizes two cameras: one for tracking which has a wide field of view, and one for inspection with a reduced field of view, allowing capturing the details and observing the elements very finely. With a displacement speed of 250 mm/s, we can control 2 elements every 30 s. The distance between camera and inspected element has been set at 600 mm with a tolerance of ±6 mm.

**Figure 28:**
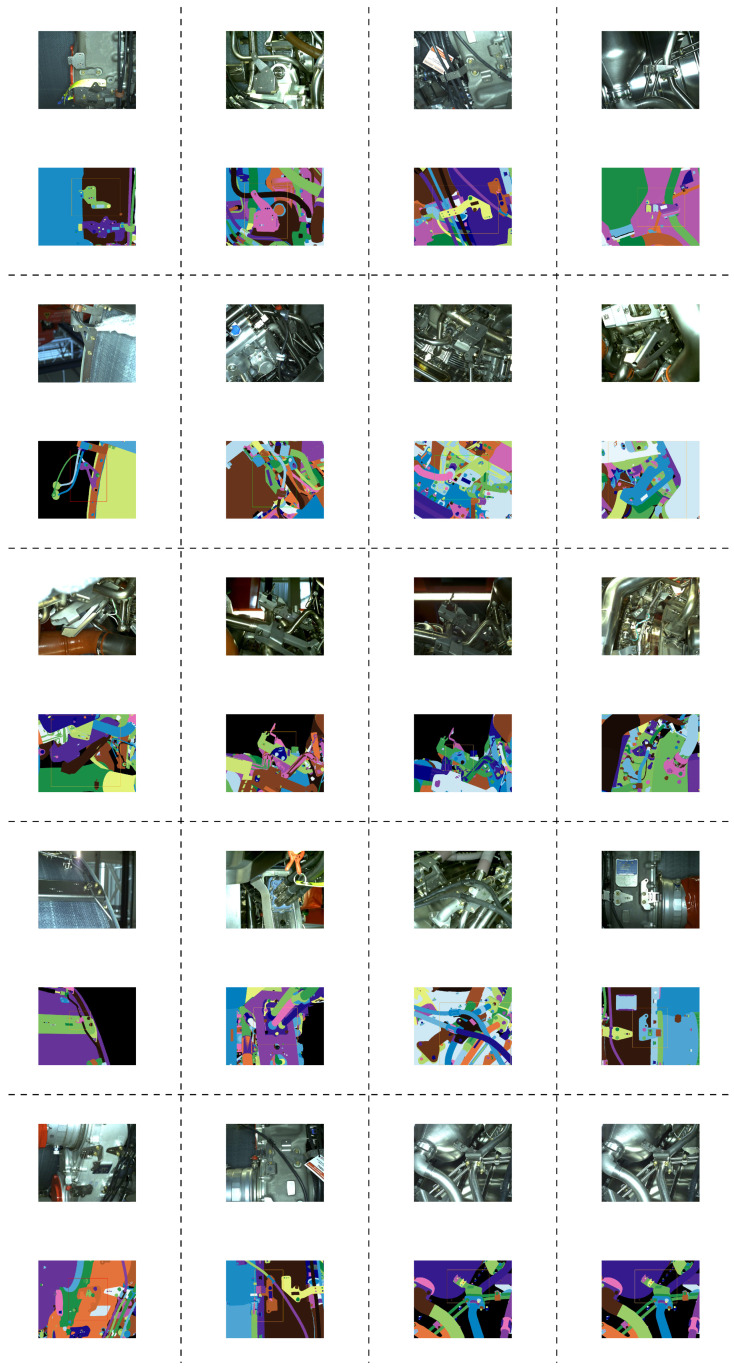
Some examples of our dataset used to evaluate our approach in a context of robotized inspection in conditions of very cluttered environment.

The approach was tested on a dataset acquired in a factory with a total number of 43 inspected elements and 643 images. Each element is acquired once at a time and decision is made on one acquired image. A few examples of our dataset are shown in Figure 28.

The overall performance of our method is represented by $F_{score}=76.72\%$. The experimental results show that our proposed approach is accurate and promising for industrial deployment. The values of specificity, accuracy, sensitivity, precision, and recall of our method are shown in Table 8 and Table 9.

Note that the results obtained with the handheld mode are better than those obtained with the robotic platform. There are three main reasons for this:

1. The robotic platform dataset is acquired in a factory, on a real and very complex assembly at the very end of the production line (almost all the elements are mounted), see Figure 28. Therefore, the environment around inspected element is very cluttered and the matching task becomes much more challenging. On the other hand, the “handheld” dataset has been acquired on quite simplified assemblies in laboratory conditions, see Figure 27. In those examples, inspected elements are mainly placed in the middle of uniform regions and parasitic edges are not many.
2. In this moment, multiview inspection is possible with handheld platform only. This means that we make a final decision on the element presence by fusing results obtained from multiple views; whereas, in robotic mode, one-shot (one view) inspection is performed. This is a feature to be added to our robotic platform as further work.
3. Mobility of the handheld system enables much more precise tracking and pose estimation which is a crucial precondition of our method.

### 6.7. Viewpoint Selection

**Figure 29:**
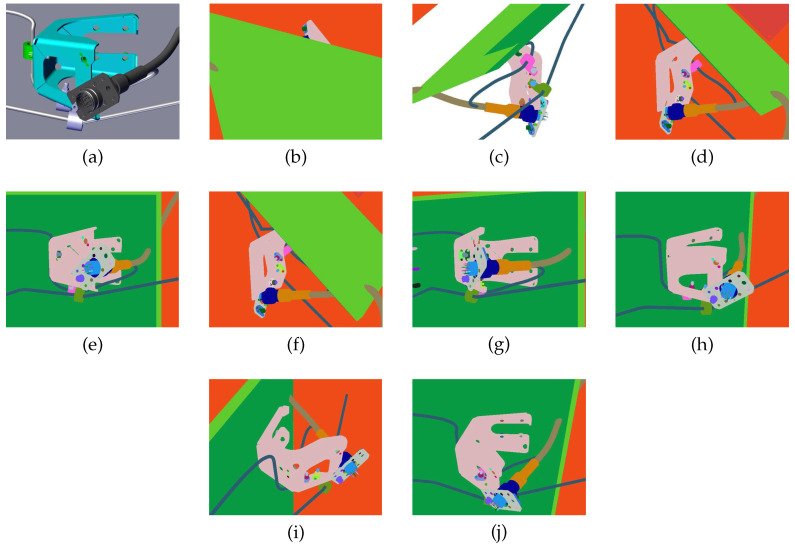
Images (**b**–**j**) correspond to different viewpoints of the element shown on image (**a**). The $f_{score}$ of each viewpoint is provided in Table 10.

In Figure 29 and Table 10, we show the result of the viewpoint selection method on an example where the weighting coefficients have been set to $w_{v}=0.5$, $w_{p}=0.25$, and $w_{s}=0.25$. The definition of additional rules for selecting automatically the weighting coefficients is under study.

## 7. Conclusions and Future Work

In this paper, we proposed an automated inspection system for mechanical assemblies in an aeronautical context. We address the problem of inspection in two parts: the first being automatic selection of informative viewpoints before the inspection process is started, and the second being automatic treatment of the acquired images from said viewpoints by matching them with information in 3D CAD model. The system is not limited to aeronautical parts; it could also be applied in a wide range of industries. The developed system shows that the precise state-of-the-art localization methods can support an industrial process. Furthermore, the experimental results show that our proposed method is highly accurate in detecting missing or badly mounted mechanical parts.

We are currently working on (1) utilizing the 3D cloud of points provided by the 3D scanner (robotized platform) in order to solve some inspection problems that cannot be solved using 2D images (for instance when we need to check that 2 cables are at a minimum distance) [21], (2) considering how the combination of 3D point clouds and 2D images can improve the detection results, (3) concerning the viewpoint selection we are trying to define some rules for choosing the weighting factors automatically, and (4) implementing multiview inspection in the robotic inspection mode in order to take the decision by fusing results obtained from multiple views.

We are also investigating deep learning-based methods relying on synthetic data that will be presented in a forthcoming paper. 

## Figures and Tables

**Table 1:** Inspection camera specifications.

Camera name	UI-3180 (PYTHON 5000)
Frame rate	73 fps
Resolution (h×v)	2592×2048
Optical Area	12.44 mm × 9.830 mm
Resolution	5.31 MPix
Pixel size	4.80 μm
Lens name	vs-2514h1
Focal length	25 mm (25.42)
Minimal object distance (M.O.D.)	300 mm
Angel of view (h×v)	$21.5^{\circ }\times 28.5^{\circ }$

**Table 2:** Tracking camera specifications.

Camera name	IDS 3070
Frame rate	123 fps
Resolution (h×v)	2056×1542
Optical Area	7.093 mm × 5.0320 mm
Resolution	3.17 MPix
Pixel size	3.45 μm
Lens name	Kowa 8 mm
Focal length	8 mm
Minimal object distance (M.O.D.)	0.1 mm
Angel of view (h×v)	$79.7^{\circ }\times 63.0^{\circ }$

**Table 3:** Determination of optimal parameter for making decision on state of element.

Inspecting Element	State of Element	Max Matched Edgelet Ratio	Min Matched Edgelet Ratio
Part A	NOK	25%	0%
Part B	OK	100%	68%
Part C	NOK	30%	0%
Part D	OK	100%	70%
Part E	OK	100%	65%
Part F	NOK	25%	0%

**Table 4:** The proportional coefficient of high and low thresholds of Canny algorithm in Figure 26a.

Figure	Figure 26b	Figure 26c	Figure 26d	Figure 26e	Figure 26f	Figure 26g	Figure 26h
($t_{l}$, $t_{h}$)	(10, 30)	(20, 60)	(30, 90)	(40, 120)	(50, 150)	(60, 180)	(70, 210)

**Table 5:** Definitions of TP, FP, TN, and FN in defect detection.

	Actually Defective	Actually Non-Defective
Detected as defective	TP	FP
Detected as nondefective	FN	TN

**Table 6:** Result of the evaluation (TP, TN, FP, and FN).

Experiment	Number of Elements	Number of Images	TP	TN	FP	FN
1 (Figure 27a,b)	3	249	114	135	0	0
2 (Figure 27c,d)	16	1580	305	1264	11	0
3 (Figure 27e,f)	3	292	193	99	0	0
4 (Figure 27g,h)	52	5135	607	4482	46	0
Total	74	7256	1219	5980	57	0

**Table 7:** Performance measures (Section 6.5).

Accuracy	Sensitivity	Specificity	Precision	Recall	$F_{\mathrm{score}}$
99.12%	100%	99.01%	95.53%	100%	97.71%

**Table 8:** Result of the evaluation (TP, TN, FP, and FN).

Number of Elements	Number of Images	TP	TN	FP	FN
43	643	145	410	88	0

**Table 9:** Performance measures (Section 6.6).

Accuracy	Sensitivity	Specificity	Precision	Recall	$F_{\mathrm{score}}$
86.31%	100%	82.32%	62.23%	100%	76.72%

**Table 10:** The $f_{score}$ of viewpoints for inspecting the element of Figure 29a.

Figure	Figure 29b	Figure 29c	Figure 29d	Figure 29e	Figure 29f	Figure 29g	Figure 29h	Figure 29i	Figure 29j
$f_{score}$	0.03766	0.16894	0.27447	0.469885	0.53009	0.55625	0.60178	0.73831	0.80842
